# Supplementary material for: Molecular architecture of meiotic pro-crossover factor HEI10 reveals coupling of higher-order assembly and ubiquitin chain formation
Source: bioRxiv. 2026 May 10:2026.05.08.723602. Preprint. [Version 1] doi: 10.64898/2026.05.08.723602 (PMC13174674; doi:10.64898/2026.05.08.723602)
Supplement: Supplement 1 [file NIHPP2026.05.08.723602v1-supplement-1.pdf]

bioRxiv preprint doi: https://doi.org/10.64898/2026.05.08.723602; this version posted May 10, 2026. The copyright holder for this preprint (which was not certified by peer review) is the author/funder, who has granted bioRxiv a license to display the preprint in perpetuity. It is made available under aCC-BY 4.0 International license.

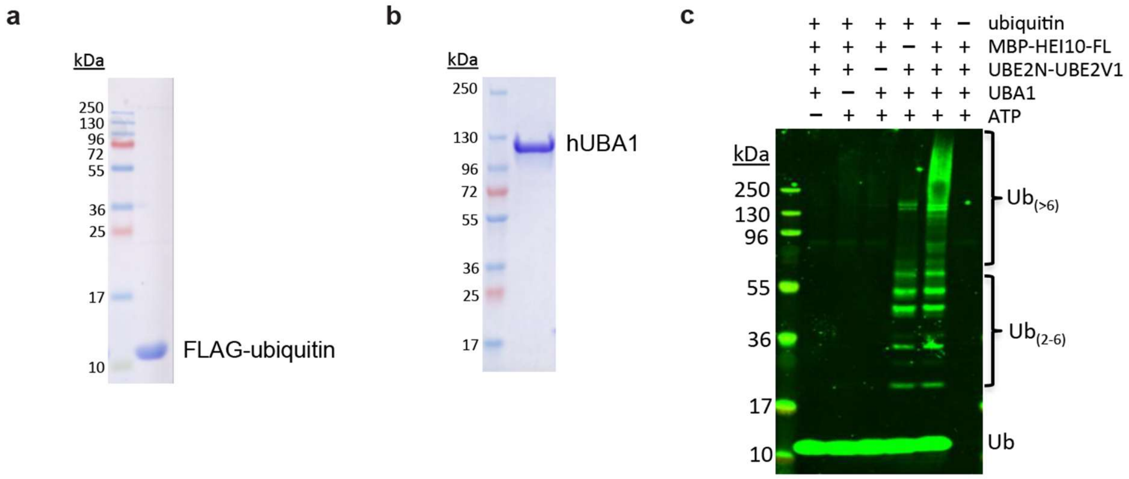

Supplementary Figure 1

HEI10 catalyses ubiquitin chain formation *in vitro*.

(**a,b**) SDS-PAGE analysis of purified FLAG-ubiquitin and human UBA1 (Boston Biochem) used in ubiquitylation reactions. (**c**) Western blot image of control ubiquitylation reactions showing that high-molecular weight $Ub_{(>6)}$ chains require all reaction components.

bioRxiv preprint doi: https://doi.org/10.64898/2026.05.08.723602; this version posted May 10, 2026. The copyright holder for this preprint (which was not certified by peer review) is the author/funder, who has granted bioRxiv a license to display the preprint in perpetuity. It is made available under aCC-BY 4.0 International license.

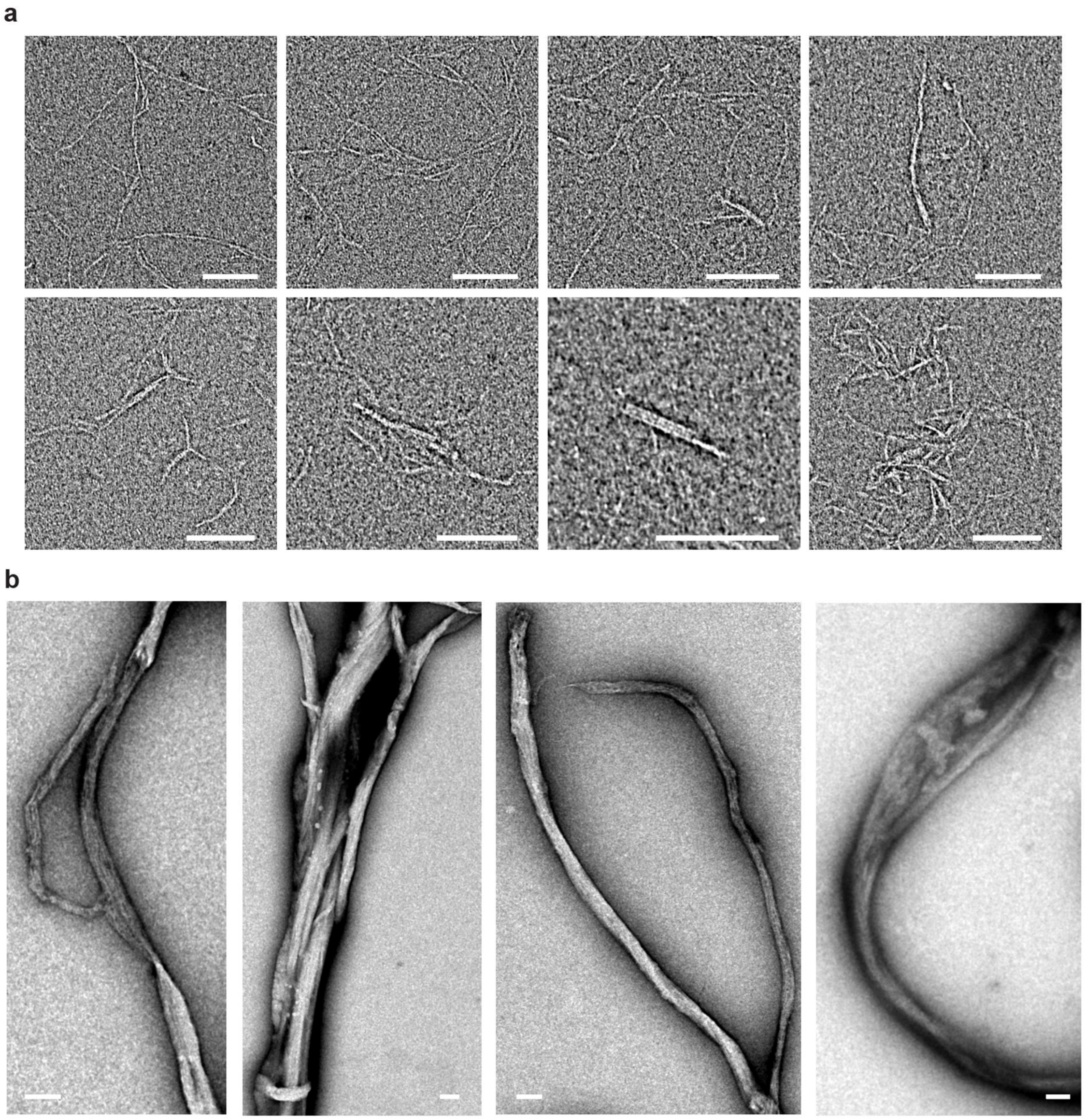

Supplementary Figure 2

Fibrous self-assembly of HEI10 *in vitro*.

Additional electron micrographs of HEI10 (full-length) pellets following TEV-cleavage from its N-terminal MBP tag, showing (**a**) internal fibrillar structure within HEI10 fibres and (**b**) large fibrous assemblies, supporting data shown in Figure 1e,f. Scale bars, 100 nm.

bioRxiv preprint doi: https://doi.org/10.64898/2026.05.08.723602; this version posted May 10, 2026. The copyright holder for this preprint (which was not certified by peer review) is the author/funder, who has granted bioRxiv a license to display the preprint in perpetuity. It is made available under aCC-BY 4.0 International license.

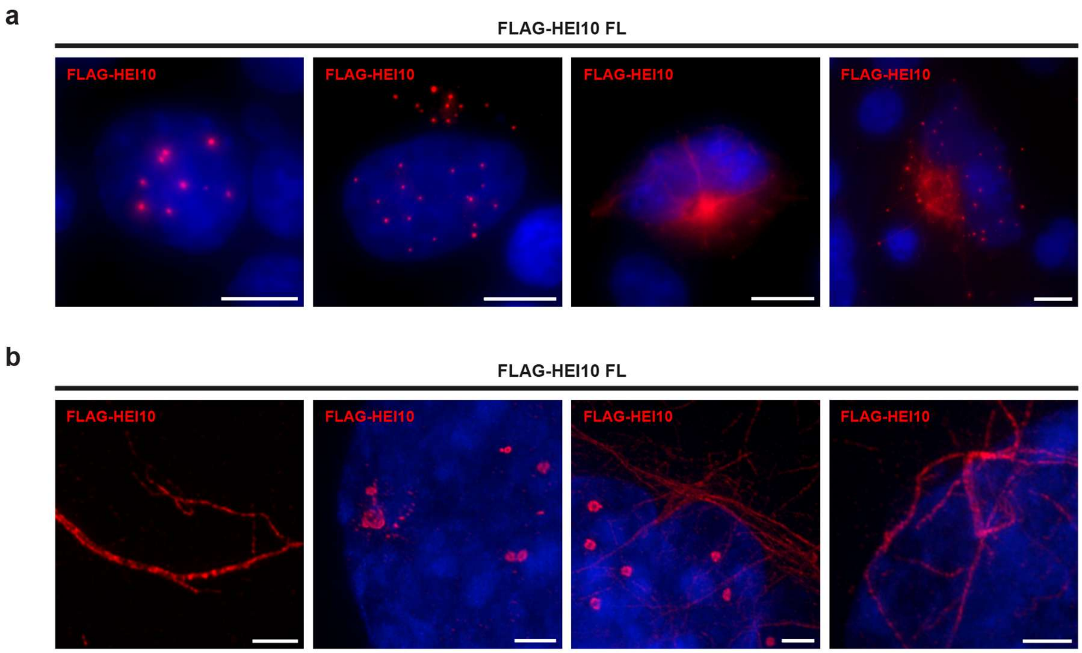

Supplementary Figure 3

HEI10 forms cytoplasmic fibres and nuclear/cytoplasmic foci upon cellular expression.

(**a,b**) Additional replicates of Flag-HEI10 over-expression in COS7 cells, with staining for Flag-HEI10 (red) and Hoechst 33342 (blue), supporting data shown in Figure 2a,b. (**a**) Wide-field imaging, showing that Flag-HEI10 forms cytoplasmic fibres and nuclear foci. Scale bars: 10 μm. (**b**) SoRa super-resolution imaging of cytoplasmic fibres (left) and nuclear foci (right). Scale bars: 2 μm.

bioRxiv preprint doi: https://doi.org/10.64898/2026.05.08.723602; this version posted May 10, 2026. The copyright holder for this preprint (which was not certified by peer review) is the author/funder, who has granted bioRxiv a license to display the preprint in perpetuity. It is made available under aCC-BY 4.0 International license.

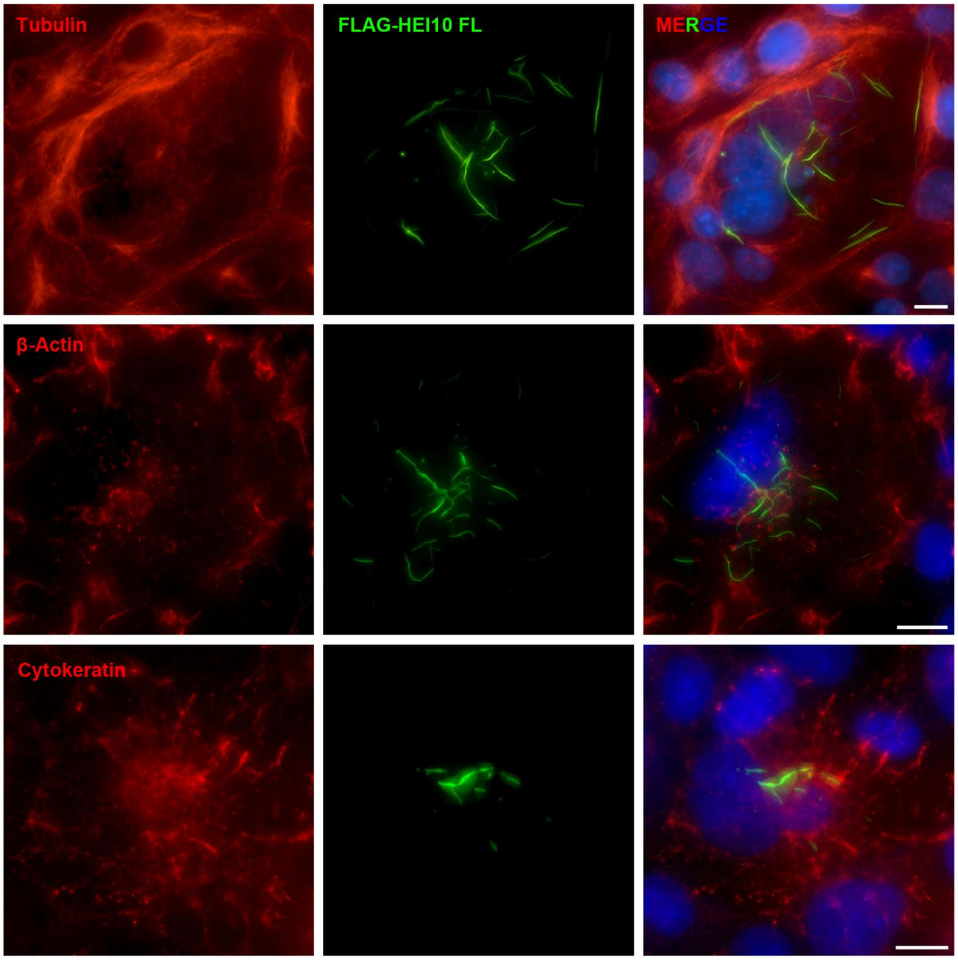

Supplementary Figure 4

Fibrous assembly of HEI10 upon expression in somatic cells.

Transient over-expression of Flag-HEI10 in COS7 cells, analysed by wide-field imaging, co-stained for Flag-HEI10 (red), DAPI, and cytoskeletal components tubulin, β-actin and cytokeratin. Scale bars, 10 μm.

bioRxiv preprint doi: https://doi.org/10.64898/2026.05.08.723602; this version posted May 10, 2026. The copyright holder for this preprint (which was not certified by peer review) is the author/funder, who has granted bioRxiv a license to display the preprint in perpetuity. It is made available under aCC-BY 4.0 International license.

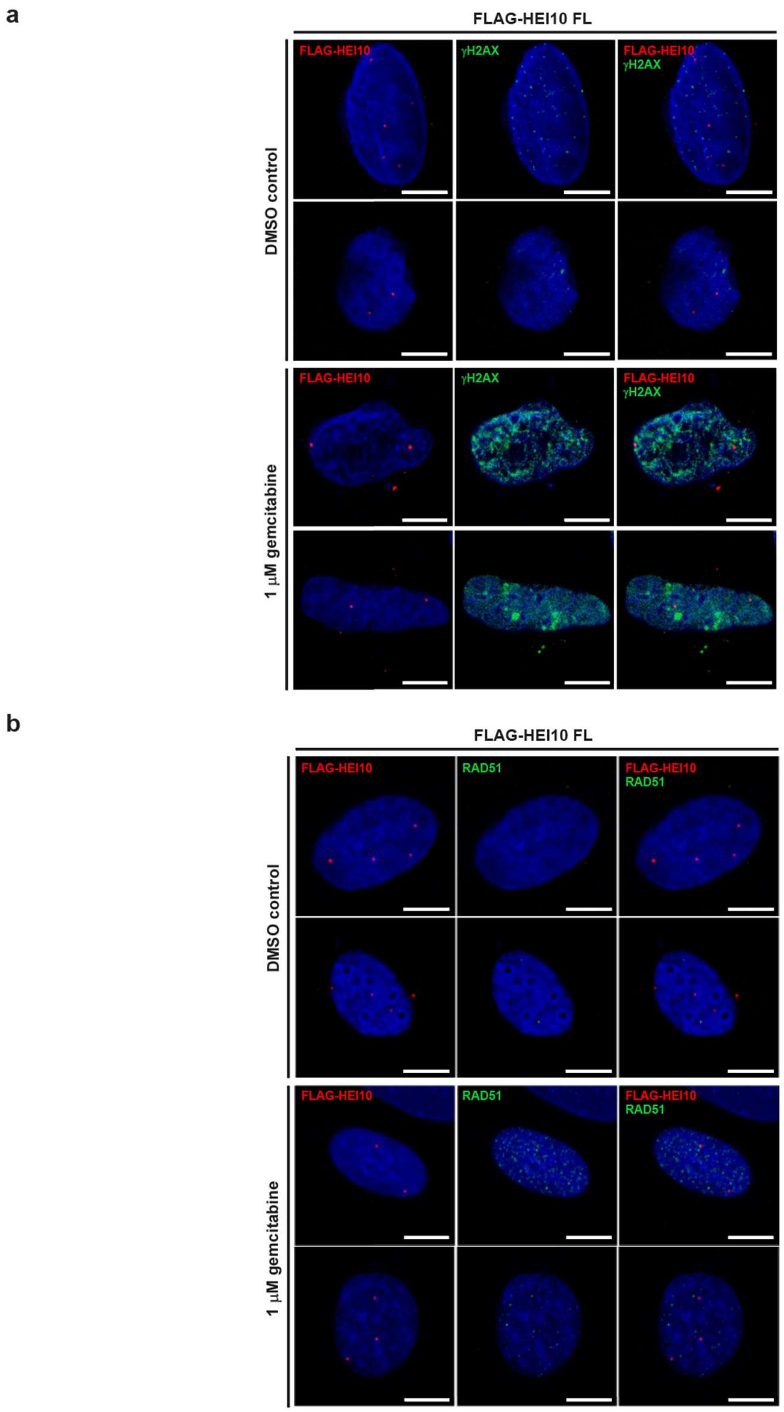

Supplementary Figure 5

HEI10 foci do not co-localise with DNA double-strand breaks.

(**a,b**) Additional replicates of Flag-HEI10 over-expression in U2OS cells, showing co-staining of Flag-HEI10 (red), with (**a**) γH2AX (green) and (**b**) RAD51 (green), alongside DAPI (blue), in cells treated with a DMSO control and 1 μM gemcitabine, supporting data shown in Figure 2c,d. Scale bars, 10 μm.

bioRxiv preprint doi: https://doi.org/10.64898/2026.05.08.723602; this version posted May 10, 2026. The copyright holder for this preprint (which was not certified by peer review) is the author/funder, who has granted bioRxiv a license to display the preprint in perpetuity. It is made available under aCC-BY 4.0 International license.

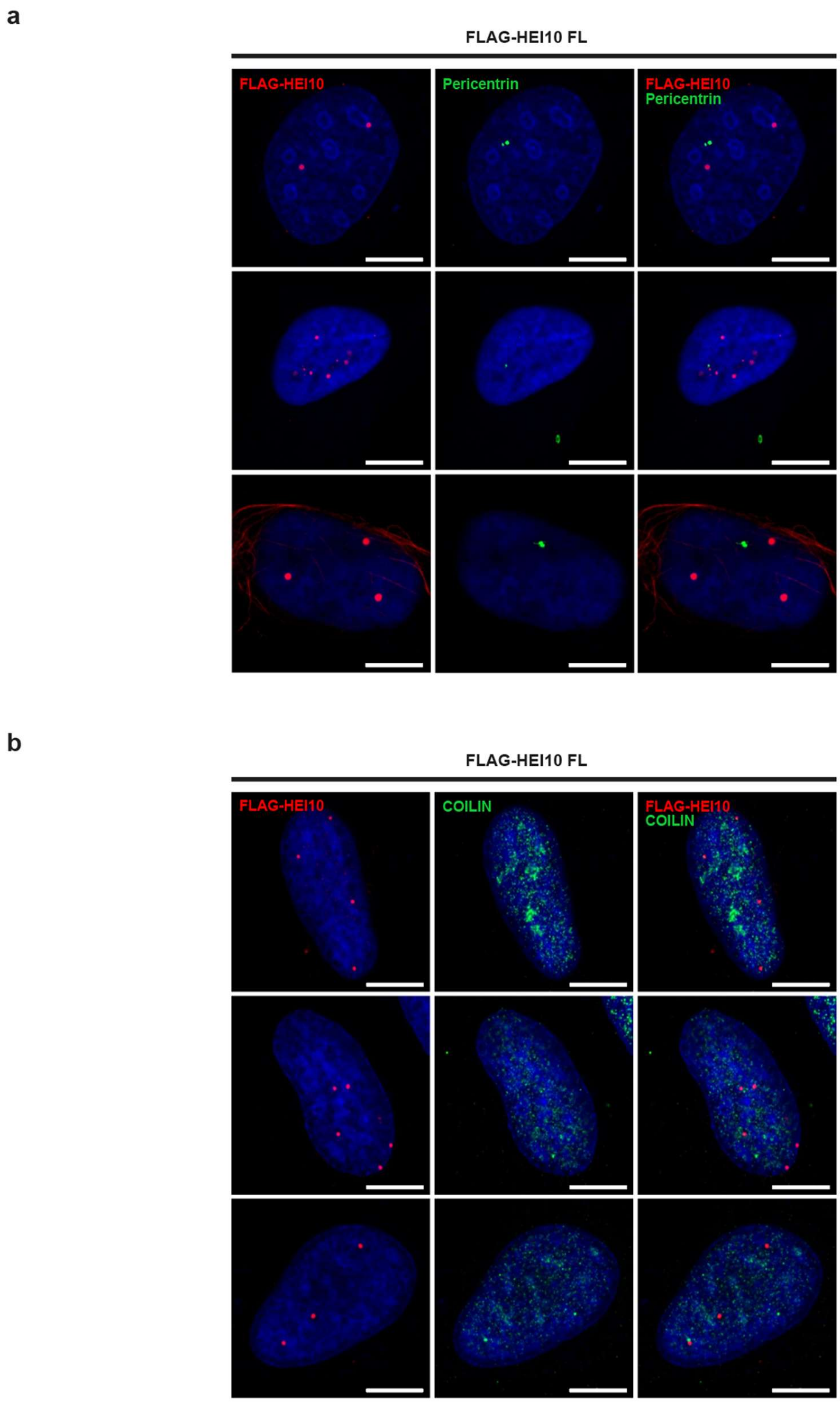

Supplementary Figure 6

HEI10 foci do not co-localise with centrosomes or Cajal bodies.

(**a,b**) Flag-HEI10 over-expression in U2OS cells. Co-staining of Flag-HEI10 (red), DAPI (blue) and (**a**) pericentrin (green) and (**b**) COILIN (green). Scale bars, 10 μm.

bioRxiv preprint doi: https://doi.org/10.64898/2026.05.08.723602; this version posted May 10, 2026. The copyright holder for this preprint (which was not certified by peer review) is the author/funder, who has granted bioRxiv a license to display the preprint in perpetuity. It is made available under aCC-BY 4.0 International license.

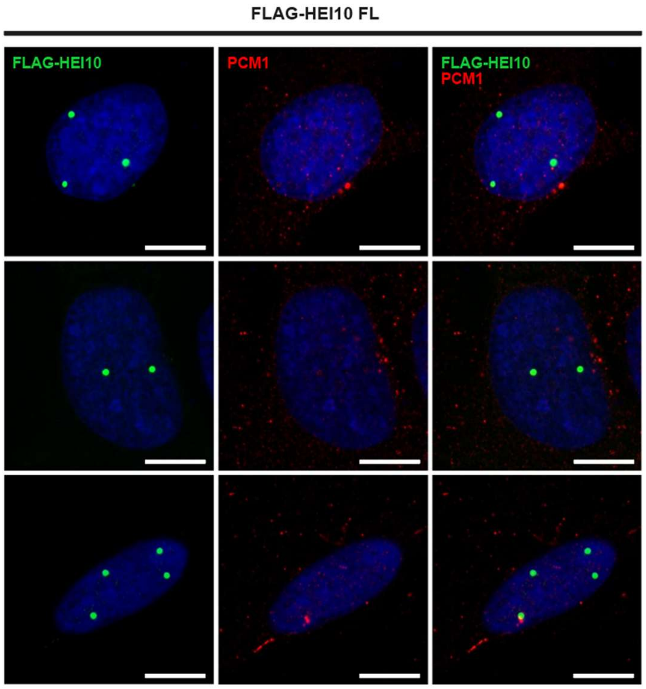

Supplementary Figure 7

HEI10 foci do not co-localise with peri-centriolar material.

Flag-HEI10 over-expression in U2OS cells. Co-staining of Flag-HEI10 (red), DAPI (blue) and PCM1 (green). Scale bars, 10 μm.

bioRxiv preprint doi: https://doi.org/10.64898/2026.05.08.723602; this version posted May 10, 2026. The copyright holder for this preprint (which was not certified by peer review) is the author/funder, who has granted bioRxiv a license to display the preprint in perpetuity. It is made available under aCC-BY 4.0 International license.

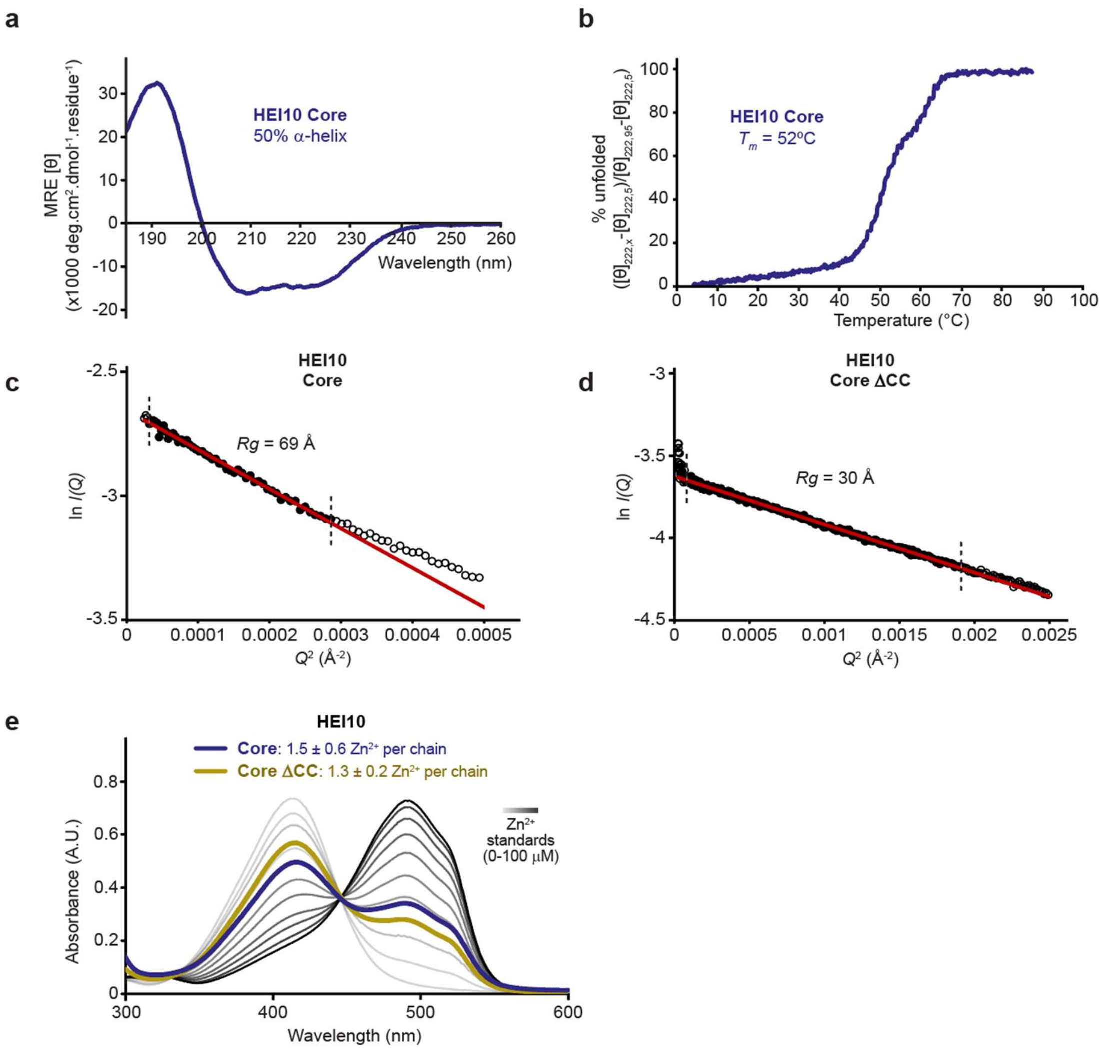

Supplementary Figure 8

HEI10 core is a stable homo-tetramer in solution that corresponds to its crystal structure.

(**a**,**b**) Circular dichroism (CD) analysis of HEI10 core. (**a**) Far UV CD spectra recorded between 260 nm and 185 nm in mean residue ellipticity, MRE ([θ]) (x10^3 deg.cm^2.dmol^{-1}.residue^{-1}). Data were deconvoluted using the CDSSTR algorithm, determining a helical content of 50%. (**b**) CD thermal denaturation of HEI10 core, recording the CD helical signature at 222 nm between 5°C and 95°C as % unfolded, indicating a melting temperature of 52°C. (**c**,**d**) SAXS Guinier analysis to determine the radius of gyration (*Rg*); linear fits are shown in red, with the fitted data range highlighted in black and demarcated by dashed lines. The *Q.Rg* values were < 1.3 and *Rg* was calculated for (**c**) HEI10 core and (**d**) HEI10 ΔCC as 69 Å and 30 Å, respectively. Corresponding to SEC-SAXS data shown in Figure 3c,d.

bioRxiv preprint doi: https://doi.org/10.64898/2026.05.08.723602; this version posted May 10, 2026. The copyright holder for this preprint (which was not certified by peer review) is the author/funder, who has granted bioRxiv a license to display the preprint in perpetuity. It is made available under aCC-BY 4.0 International license.

(**e**) Spectrophotometric determination of zinc content for HEI10 core (dark blue; 1.5 ± 0.6 Zn^{2+} per chain, n = 6) and HEI10 1-122 (yellow; 1.3 ± 0.2 Zn^{2+} per chain, n = 3), using metallochromic indicator PAR, with zinc standards shown in a gradient from light to dark grey (0-100 µM) and proteins analysed at 20 µM (per chain).

bioRxiv preprint doi: https://doi.org/10.64898/2026.05.08.723602; this version posted May 10, 2026. The copyright holder for this preprint (which was not certified by peer review) is the author/funder, who has granted bioRxiv a license to display the preprint in perpetuity. It is made available under aCC-BY 4.0 International license.

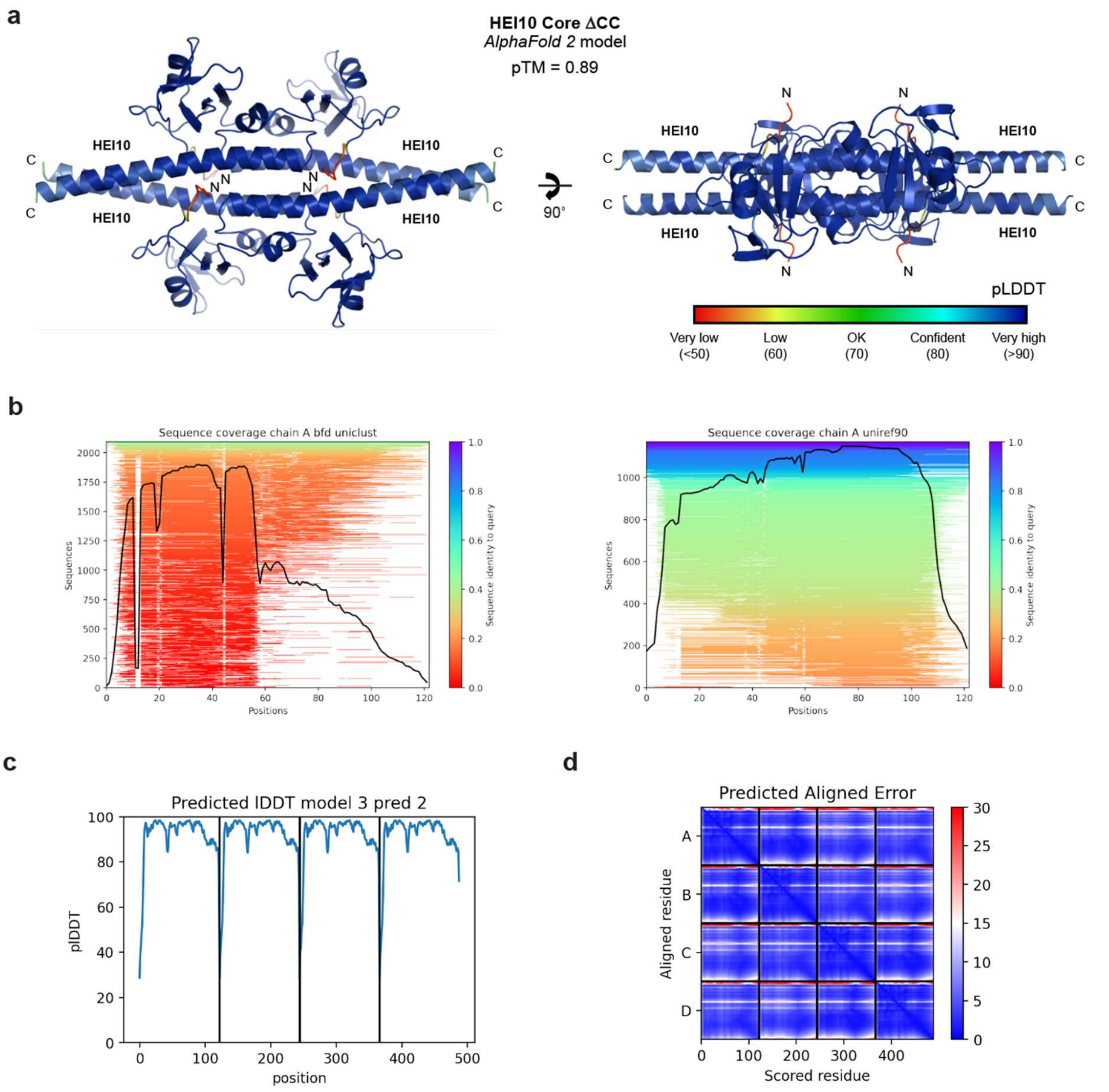

Supplementary Figure 9

***AlphaFold 2* model of the HEI10 core ΔCC tetramer.**

(**a**) *AlphaFold 2* model of the HEI10 core ΔCC tetramer coloured according to predicted LDDT (pLDDT) scores, between blue (>90) and red (<50). (**b**) Representations of the multiple sequence alignments generated and used by *AlphaFold 2*, showing the number of sequences and sequence identity against the position along the HEI10 sequence. (**c**) Predicted LDDT (pLDDT) scores shown for each amino-acid of the four HEI10 chains. (**e**) Predicted aligned error scores between each amino-acid of the four HEI10 chains, between blue (low error) and red (high error).

bioRxiv preprint doi: https://doi.org/10.64898/2026.05.08.723602; this version posted May 10, 2026. The copyright holder for this preprint (which was not certified by peer review) is the author/funder, who has granted bioRxiv a license to display the preprint in perpetuity. It is made available under aCC-BY 4.0 International license.

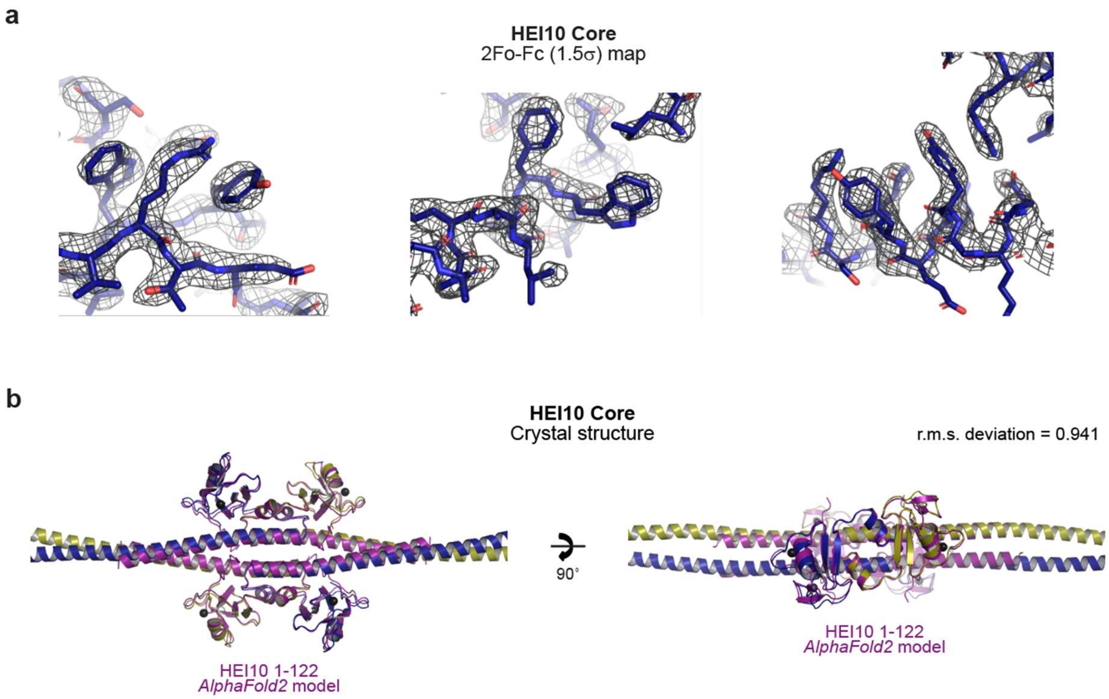

Supplementary Figure 10

Crystal structure of HEI10 core.

(**a**) 2Fo-Fc electron density map of the HEI10 core structure, contoured at 1.5σ, superimposed on the refined crystallographic model. (**b**) Superposition of the HEI10 core crystal structure (yellow and blue) with the HEI10 core ΔCC *AlphaFold 2* model (purple), showing an r.m.s. deviation of 0.941.

bioRxiv preprint doi: https://doi.org/10.64898/2026.05.08.723602; this version posted May 10, 2026. The copyright holder for this preprint (which was not certified by peer review) is the author/funder, who has granted bioRxiv a license to display the preprint in perpetuity. It is made available under aCC-BY 4.0 International license.

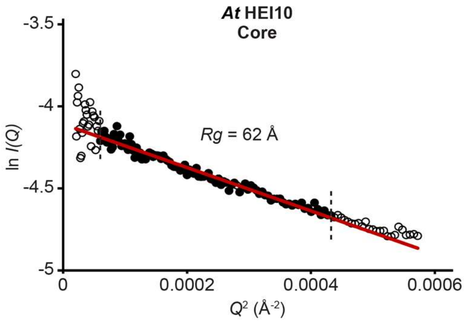

Supplementary Figure 11

SEC-SAXS analysis of *At* HEI10 core.

SAXS Guinier analysis to determine the radius of gyration (*Rg*); linear fits are shown in red, with the fitted data range highlighted in black and demarcated by dashed lines. The *Q*.*Rg* values were < 1.3 and *Rg* was calculated as 62 Å. Corresponding to SEC-SAXS data shown in Figure 6d,e.

bioRxiv preprint doi: https://doi.org/10.64898/2026.05.08.723602; this version posted May 10, 2026. The copyright holder for this preprint (which was not certified by peer review) is the author/funder, who has granted bioRxiv a license to display the preprint in perpetuity. It is made available under aCC-BY 4.0 International license.

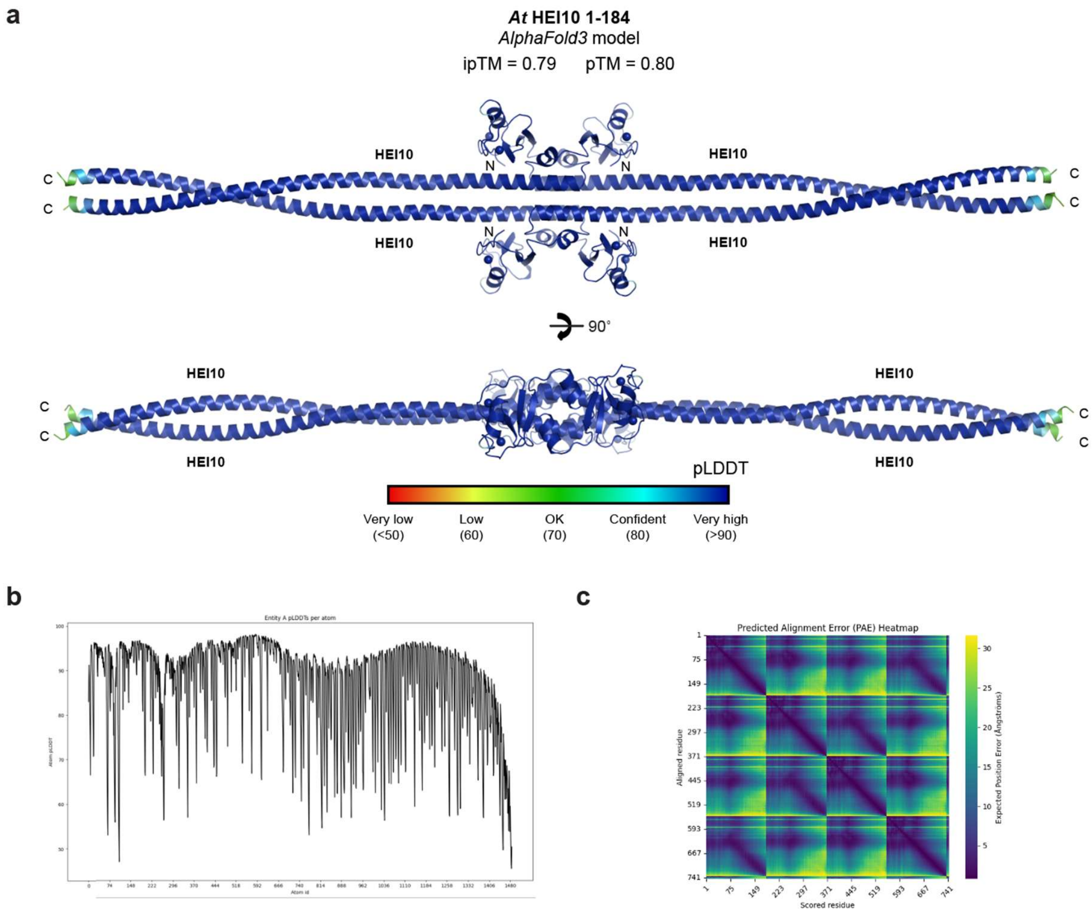

Supplementary Figure 12

***AlphaFold 3* model of the *At* HEI10 core tetramer.**

(**a**) *AlphaFold 3* model of the *At* HEI10 core tetramer coloured according to predicted LDDT (pLDDT) scores, between blue (>90) and red (<50). (**b**) Predicted LDDT (pLDDT) scores shown for each amino-acid of the four *At* HEI10 chains. (**c**) Predicted aligned error scores between each amino-acid of the four *At* HEI10 chains, between blue (low error) and yellow (high error).

bioRxiv preprint doi: https://doi.org/10.64898/2026.05.08.723602; this version posted May 10, 2026. The copyright holder for this preprint (which was not certified by peer review) is the author/funder, who has granted bioRxiv a license to display the preprint in perpetuity. It is made available under aCC-BY 4.0 International license.

a

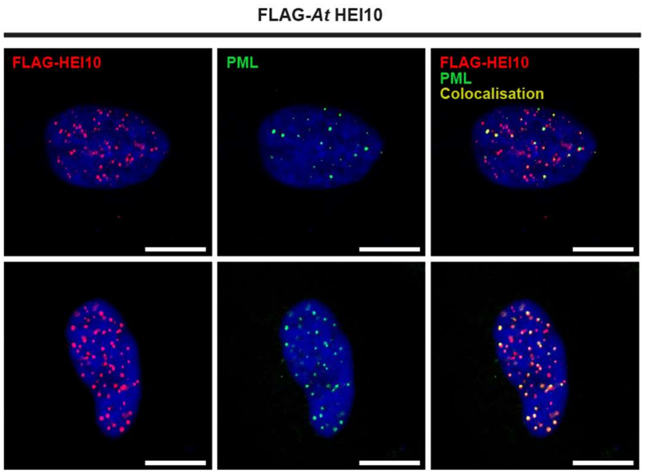

b

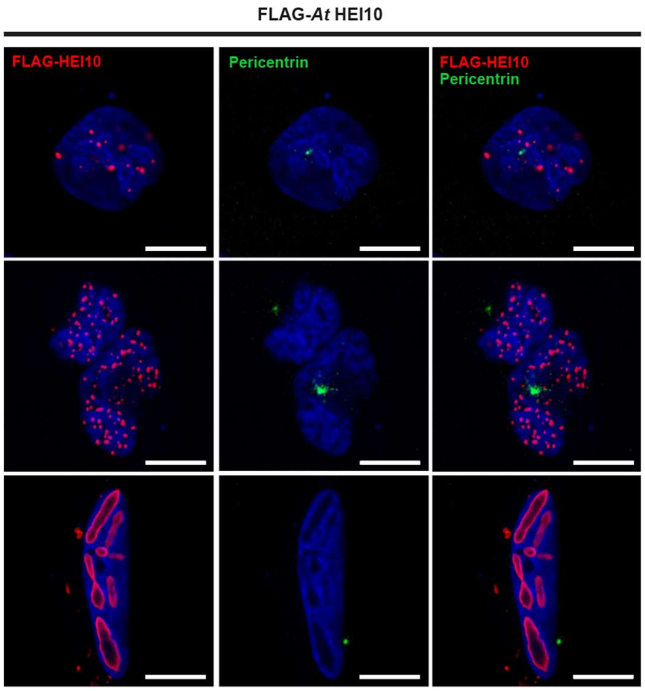

Supplementary Figure 13

***At* HEI10 foci localise with PML bodies but not centrosomes.**

(**a**,**b**) Flag-*At* HEI10 over-expression in U2OS cells. Co-staining of Flag-*At* HEI10 (red), DAPI (blue) and (**a**) PML (green) and (**b**) pericentrin (green), supporting data in Figure 6g. Scale bars, 10 μm.

bioRxiv preprint doi: https://doi.org/10.64898/2026.05.08.723602; this version posted May 10, 2026. The copyright holder for this preprint (which was not certified by peer review) is the author/funder, who has granted bioRxiv a license to display the preprint in perpetuity. It is made available under aCC-BY 4.0 International license.

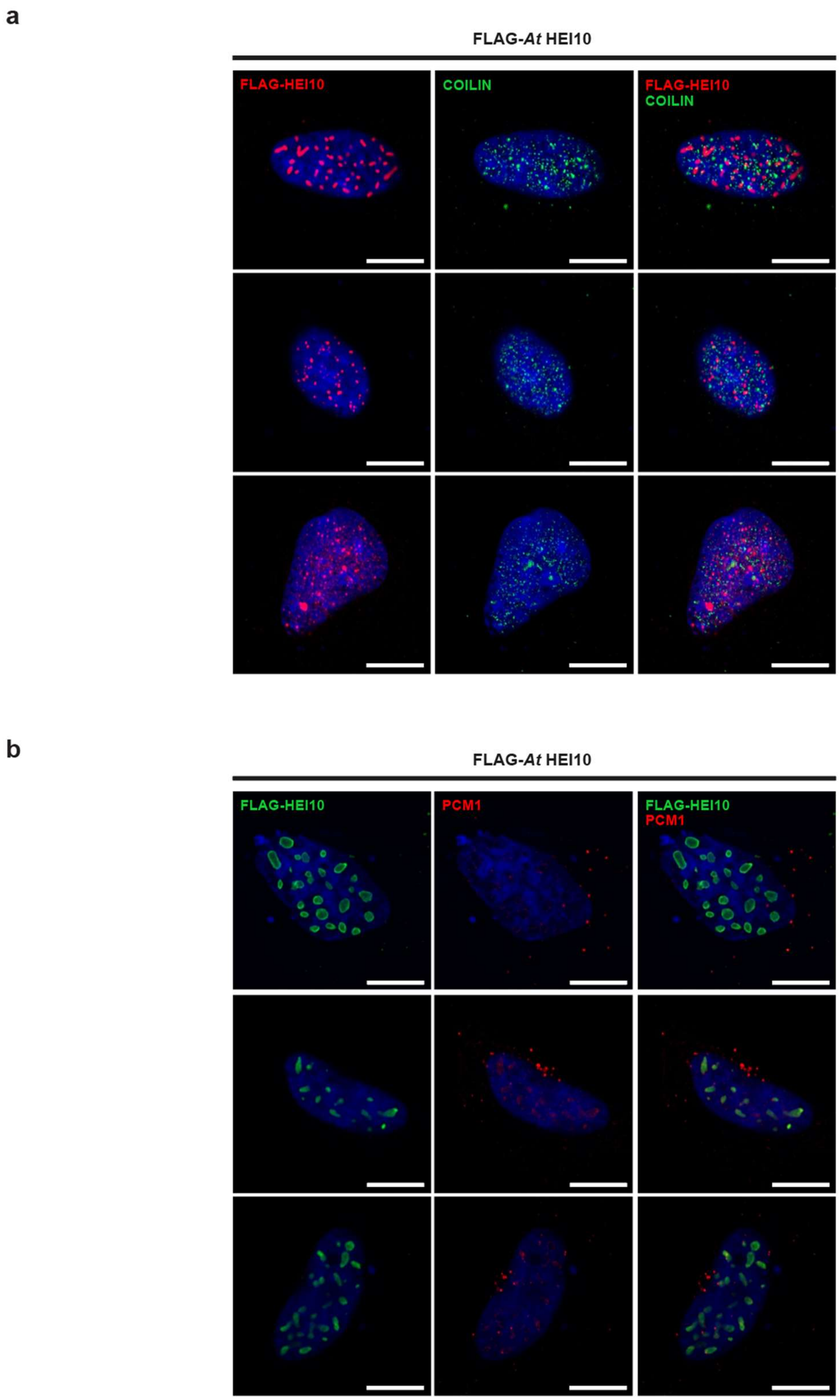

Supplementary Figure 14

***At* HEI10 foci do not localise with Cajal bodies or peri-centriolar material.**

(**a**,**b**) Flag-*At* HEI10 over-expression in U2OS cells. Co-staining of Flag-*At* HEI10 (red), DAPI (blue), and (**a**) COILIN (green) and (**b**) PCM1 (green). Scale bars, 10 μm.

bioRxiv preprint doi: https://doi.org/10.64898/2026.05.08.723602; this version posted May 10, 2026. The copyright holder for this preprint (which was not certified by peer review) is the author/funder, who has granted bioRxiv a license to display the preprint in perpetuity. It is made available under aCC-BY 4.0 International license.

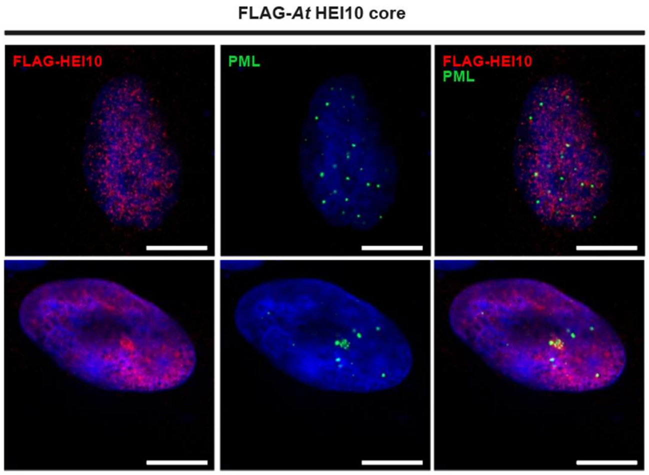

Supplementary Figure 15

***At* HEI10 core foci do not localise with PML bodies.**

Flag-*At* HEI10 over-expression in U2OS cells. Co-staining of Flag- *At* HEI10 (red) PML1 (green) and DAPI (blue), supporting data in Figure 6h. Scale bars, 10 μm.
